# Theory of partial agonist activity of steroid hormones

**DOI:** 10.3934/molsci.2015.2.101#sthash.jxRCteJz.dpuf

**Published:** 2015

**Authors:** Carson C. Chow, Karen M. Ong, Benjamin Kagan, S. Stoney Simons

**Affiliations:** 1 Mathematical Biology Section, NIDDK/LBM, National Institutes of Health, Bethesda, MD 20892-5621, USA; 2 Steroid Hormones Section, NIDDK/LERB, National Institutes of Health, Bethesda, MD 20892-1772, USA; 3 Computational Biology Program, New York University School of Medicine, New York, NY 10016, USA; 4 Science Department, Tuscarora High School, Loudoun County Public Schools, Leesburg, VA 20176, USA

**Keywords:** antisteroid, antagonist, antiestrogen, tamoxifen, raloxifene, ligand, nuclear receptor, steroid receptor, glucocorticoid receptor, gene expression, gene transcription

## Abstract

The different amounts of residual partial agonist activity (PAA) of antisteroids under assorted conditions have long been useful in clinical applications but remain largely unexplained. Not only does a given antagonist often afford unequal induction for multiple genes in the same cell but also the activity of the same antisteroid with the same gene changes with variations in concentration of numerous cofactors. Using glucocorticoid receptors as a model system, we have recently succeeded in constructing from first principles a theory that accurately describes how cofactors can modulate the ability of agonist steroids to regulate both gene induction and gene repression. We now extend this framework to the actions of antisteroids in gene induction. The theory shows why changes in PAA cannot be explained simply by differences in ligand affinity for receptor and requires action at a second step or site in the overall sequence of reactions. The theory also provides a method for locating the position of this second site, relative to a concentration limited step (CLS), which is a previously identified step in glucocorticoid-regulated transactivation that always occurs at the same position in the overall sequence of events of gene induction. Finally, the theory predicts that classes of antagonist ligands may be grouped on the basis of their maximal PAA with excess added cofactor and that the members of each class differ by how they act at the same step in the overall gene induction process. Thus, this theory now makes it possible to predict how different cofactors modulate antisteroid PAA, which should be invaluable in developing more selective antagonists.

## 1. Introduction

Antisteroids, antagonists, or partial agonists are defined as ligands that compete with agonist steroid hormones for the binding to cognate receptor proteins to evoke reduced or no biological activity. Initially, the most desirable antisteroids were those that completely blocked the action of agonist steroids by one of a variety of possible mechanisms. However, it gradually became apparent that the most clinically useful antisteroids would be those that blocked the activity of agonist steroids with one particular target gene while sparing other target genes [1,2,3]. In this manner, the “off-target” actions, or side effects, can be minimized. Unfortunately, it has proved difficult to predict the overall activities. For example, the antiestrogens raloxifene and tamoxifen are equally effective in preventing the growth of estrogen-dependent breast cancers without inhibiting bone responses but raloxifene has much reduced estrogenic effects in uteri, making it preferable in treating breast cancers [1,4]. Similarly, the antiglucocorticoid C108297 is of interest because it displays agonist or antagonist activity in rat brains depending on the gene or neurological function monitored [5].

A necessary characteristic of antisteroids with mixed activities, like raloxifene and C108297, is that they display significant amounts of agonist activity with some target genes. For this reason, this group of antagonists is often called “selective receptor modulators” (SRMs), and more specifically, for example, “selective estrogen receptor modulators” (SERMs) [6] or “selective glucocorticoid receptor modulators” (SGRMs) [5]. The amount of residual agonist activity of the SRM with a given reporter gene is often referred to as the partial agonist activity (PAA). If concentrations of a given SRM sufficient to saturate the binding of the intended receptor protein give 30% of the maximal activity seen with saturating concentrations of a full agonist, that SRM is said to have 30% PAA.

Before the advent of SRMs, when limited reporter genes were available and various antisteroids were found to possess different amounts of PAA, it was presumed that the value of the PAA was an invariant property of that antagonist [7]. The discovery of SRMs like tamoxifen and raloxifene discredited this hypothesis. However, it was commonly believed that, for each SRM, at least the PAA value with a given reporter gene in specific cell was a constant. Subsequent detailed studies revealed, though, that this concept too was invalid. In particular, the relative concentration of various transcription factors and cofactors, including coactivators, corepressors, and the receptors themselves, were found capable of acting like a rheostat to vary the PAA from, in some cases, close to 0 to almost 100% [2,8–13]. One might intuitively expect that the PAA would remain constant under such conditions. For example, take a partial agonist that gives 30% PAA. With the addition of a coactivator, which by definition increases the absolute activity of the agonist, one might expect that the PAA of the antisteroid would be increased proportionately to give 30% of the now larger value of the agonist steroid, so that the PAA does not change. However, this is not the case. The addition of a coactivator usually augments the activity of the antisteroid more than that of the agonist so that the PAA *increases* [2,8–13].

The mechanism underlying this disproportionate increase in activity of antisteroid versus agonist steroid, with the resulting rise in PAA, is currently obscure. One explanation derives from intrinsic, albeit unidentified, differences between the steroids themselves. In some, but not all, cases there can be a large difference in the dissociation constant for steroid binding to receptor, with the early hypothesis being that more weakly binding steroids will display less agonist activity [14]. However, this clearly cannot be a general determinant because the PAA of the SGRM and affinity label dexamethasone 21-mesylate (DM) decreases as the amount of covalent, irreversibly bound receptor-steroid complex increases [15,16]. A second explanation is that ligand-induced changes in receptor tertiary structure alter subsequent interactions of receptor-steroid complexes with cofactors to modify the efficacy of gene transcription [17–21] but there has of yet been no means to test this hypothesis directly.

Here, we construct a theory capable of explaining these experimental observations. The theory also supports the second hypothesis for partial agonist activity. The theory is based on the fact that the dose-response curve for steroid-regulated gene expression is usually non-cooperative with a Hill coefficient of one. For such regulated genes, this fact has been used to develop experimentally validated models that explain ligand-regulation of both gene induction and repression [22–28]. In view of these successes, we sought to use the same methodology to explain the actions of antagonists. Here, we show how these approaches can be extended to describe the variable properties of SRMs. The theory indicates that differences in binding affinity alone cannot account for the PAAs. Rather, the variations in activities between antagonists are due to differences in biochemical actions downstream from receptor-agonist binding. If a cofactor is known to increase the activity of a particular antagonist, then the theory predicts that all antagonists that converge to the same PAA with large amounts of that cofactor differ in their action at a single mechanistic step. This provides a novel means of classifying antagonists. Similarly incomplete with regard to mechanism is the popular explanation that the altered steroid structure of antisteroids perturbs the topology of the resulting receptor-steroid complex from that seen with agonists, thereby acting like a binary switch, with corepressors replacing the coactivators that associate with agonist-bound receptors [17,29]. Our theory offers an alternative explanation.

## 2. Materials and Methods

Unless otherwise indicated, all cell growth was at 37 °C and all other operations were performed at room temperature.

### Chemicals

Dexamethasone (Dex), 11-deoxycorticosterone [DOC], progesterone (Prog), and RU486 are from Sigma (St. Louis, MO), R5020 from PerkinElmer (Boston, MA), and dexamethasone 21-mesylate (Dex-21-mesylate; DM) from Steraloids (Newport, RI). Dexamethasone oxetanone [Dex-Ox] was prepared as described [30]. Dual-luciferase reporter assay is from Promega (Madison, WI).

### Plasmids

Rat GR (pSG5-GR), GREtkLUC, Renilla-TS [9] and pSG5/Ubc9 and pSG5/hSA [31] have been previously reported.

### Cell culture, transient transfection, and reporter analysis

Monolayer cultures of CV-1 cells were grown as described previously [32]. Triplicate samples of cells were seeded into 24-well plates at 20,000 cells per well and transiently transfected the following day with luciferase reporter and DNA plasmids by using 0.7 μL Fugene 6 (Roche) per well according to the manufacturer’s instructions. The total transfected DNA was adjusted to 300 ng/well of a 24-well plate with pBluescriptII SK+ (Stratagene). The molar amount of plasmids expressing different protein constructs was kept constant with added empty plasmid or plasmid expressing human serum albumin [9]. Renilla-TS (10 ng/well of a 24-well plate) was included as an internal control. After transfection (32 h), cells were treated with medium containing appropriate hormone dilutions. The cells were lysed 20 hr later and assayed for reporter gene activity using dual luciferase assay reagents according to the manufacturer’s instructions (Promega, Madison, WI). Luciferase activity was measured by an EG&G Berthold’s luminometer (Microlumat LB 96P). The data were normalized to Renilla TS luciferase activity and expressed as a percentage of the maximal response with Dex before being plotted ± standard error of the mean, unless otherwise noted.

### Data analysis

Each average of triplicates is treated as one value of the *n* experiments. Best-fits of the dose-response curves following first-order Hill plots (R^2^ almost always ≫ 0.95) were obtained with KaleidaGraph (Synergy Software, Reading, PA). Fits of the PAA to a linear-fractional function were performed with the *nls* package in the open source software environment *R* (R-project.org).

## 3. Results

### 3.1. Theory for partial agonist action

We define PAA as the ratio of the maximal activity, *A*_max_, of an antisteroid, antagonist or partial agonist to the maximal activity of the control agonist. We define the partial potency (PP) as the ratio of the potency, measured in terms of EC_50_, of the partial agonist to the control agonist. The EC_50_ is the concentration of ligand that gives 50% of the maximal activity with the same ligand. We define these quantities as fractions for convenience but it is trivial to convert them to percentages by multiplying by 100%. We will use the prime symbol to denote quantities related to the partial agonist. Our goal is to make theoretical predictions for PAA and PP and compare them to experimental data for various partial agonists.

We start with our previously developed theory of gene expression, which is predicated on the experimental observation that the dose-response curve of the gene product versus the ligand concentration is non-cooperative with a Hill coefficient of one, i.e., it is a Michaelis-Menten function with the form *v*[*L*]/(1+*w*[*L*]), where [*L*] is the ligand concentration, *v* is A_max_/EC_50_ and *w* is 1/EC_50_. The theory has been validated in multiple experiments and is based on the principle that steroid-regulated gene expression involves a sequence of steps that begins with the binding of steroid to receptor and ends with the gene product [22–28]. We modeled this biochemically as a sequence of complex building reactions $Y_{i-1}+X_{i}\overset{q_{i}}{↔}Y_{i}$, where the *Y* variables are called “products”, the *X* variables are called “accelerators”, and the *q* variables can be called association, equilibrium, or affinity constants [22,23]. Added transcription cofactors (coactivators or corepressors) can act either as accelerators or as decelerators that inhibit accelerators [24]. The locations of reactions can be placed with respect to each other and a distinguished step called the concentration-limited step (CLS) [22–24,27,28]. The step after the CLS acts as an equilibrium analog of a rate-limiting step and is marked by the fact that before and at the CLS, accelerators are limited, while after the CLS products are limited and accelerators are abundant.

The dose-response is the concentration of the final product of the reaction sequence as a function of the initial product (ligand) and can be explicitly calculated in the mass action limit. As we showed previously, the dose-response will be non-cooperative if downstream products have smaller concentrations than upstream ones, if product lifetimes are short, or if accelerators are abundant compared to products [22,28]. Under these conditions, an explicit formula for the dose-response can be written in terms of the affinity constants and total accelerator/decelerator concentrations of every reaction in the sequence. Even if most if not all of the parameters are unknown, the dose-response formula is useful to probe the action of a small set of specific cofactors because it has qualitatively distinct forms depending on how and where these cofactors act. In particular, the PAA and PP can be characterized by a small number of effective parameters that depend on the precise mechanism and position of action of the cofactor.

Our hypothesis for why a partial agonist has different activity from a full agonist is that the binding affinity of the components of at least one reaction downstream of ligand-receptor binding in the gene expression sequence differs between the two ligands. Using our theory, we derive formulae for PAA and PP between a partial agonist and the full agonist. We also find that the ratio of PAA to PP provides additional useful information. We consider the specific case where the two ligands give rise to the same sequence of reactions but differ in both the binding affinity between ligand and receptor and the binding affinity of at least one downstream reaction. The formulae can be easily generalized to include differences in multiple positions and include the influence of numerous accelerators and decelerators. However, as we show below, the derived formulae are sufficient to explain the available experimental data.

When the ligand-receptor binding affinity and the binding affinity of one downstream reaction at position *d* differ between a partial and full agonist, the formulae for the ratios, which are derived in full in the Appendix, can be expressed as

$$\begin{array}{c} \mathrm{PAA}=\left(\frac{C_{1}+C_{2}q_{d}^{\prime }}{C_{1}+C_{2}q_{d}}\right)\left(\frac{1+\left(C_{3}+C_{4}q_{d}\right)X_{1}^{T}}{1+\left(C_{3}+C_{4}q_{d}^{\prime }\right)X_{1}^{T}}\right)\\ PP=\left(\frac{1+\left(C_{3}+C_{4}q_{d}\right)X_{1}^{T}}{1+\left(C_{3}+C_{4}q_{d}^{\prime }\right)X_{1}^{T}}\right)\left(\frac{q_{1}}{q_{1}^{\prime }}\right)\\ \frac{\mathrm{PAA}}{PP}=\left(\frac{C_{1}+C_{2}q_{d}^{\prime }}{C_{1}+C_{2}q_{d}}\right)\left(\frac{q_{1}^{\prime }}{q_{1}}\right) \end{array}$$

where *d* is an integer corresponding to the step in the overall reaction sequence with *d* = 2 being the step after ligand binding to the receptor, $X_{1}^{T}$ is the total concentration of the receptor, *q*_1_ and $q_{1}^{\prime }$ are the respective ligand-receptor affinity constants for the full and partial agonists, *q_d_* and $q_{d}^{\prime }$ are the respective affinity constants for a downstream reaction at position *d*, and *C*_1_ through *C*_4_ are effective positive parameters that are functions of the parameters of hidden reactions. The formulae take different forms depending on where the accelerators are located with respect to each other and the CLS. With a receptor and one accelerator, there will be reactions before, after, and between the reactions, each with their own affinity constants and total accelerator concentrations. The combined effect of these hidden reactions on PAA and PP are encoded in the *C* parameters, which have different values depending context. For example, if *d* is before or at the CLS, then parameter *C*_1_ = 0, and if *d* = 2, which is the reaction immediately following ligand-receptor binding, then *C*_3_ = 0 (see Appendix).

As seen in the Appendix, the formulae for the quantities A_max_/EC_50_ and 1/EC_50_ of the dose-response are linear in the accelerator affinities and total concentrations and are thus the more mathematically convenient objects. The formula for PP is given by the ratio of the respective formulae for 1/EC_50_ while the PAA is formed by the ratio of A_max_/EC_50_ divided by the ratio of 1/EC_50_, which is why it consists of two linear-fractional factors, with the second factor arising from PP. The ratio of PAA to PP is the ratio of the respective A_max_/EC_50_’s and echoes the first factor of the PAA. One immediately sees from the formula for PAA that receptor-ligand binding affinities, *q*_1_ and $q_{1}^{\prime }$, are missing and thus do not affect PAA. This is because receptor-ligand binding affinity only affects EC_50_ and not A_max_ in the dose-response.

The values of PAA and PP depend on the parameters of the reactions and thus can be used to make predictions for the mechanisms of partial agonist activity. In the limit of no receptors, PAA is less than (greater than) 100% if $q_{d}^{\prime }<q_{d}(q_{d}^{\prime }>q_{d})$ and PP is given by the ratio of the receptor binding affinities of the two ligands. If the downstream reaction, *d*, that is different is before or at the CLS then *C*_1_ = 0 and PAA is given by the ratio of the affinities of the downstream reaction products for cofactors. Experimentally, however, extrapolating down to very low receptor numbers is confounded by the facts that the gene may have some low baseline transcription rate and that the ligands may compete with other endogenous inducers.

A more productive approach is to consider how the ratios change as we add receptor. Both PAA and PP are predicted to be linear-fractional functions of receptor concentration. PAA will increase when elevating the receptor number if $q_{d}^{\prime }<q_{d}$ and decrease if $q_{d}^{\prime }>q_{d}$. The saturated value of PAA for excessive receptor is given by

$$\left(\frac{C_{1}/C_{2}+q_{d}^{\prime }}{C_{1}/C_{2}+q_{d}})(\frac{C_{3}/C_{4}+q_{d}}{C_{3}/C_{4}+q_{d}^{\prime }}\right)$$

If *C*_1_/*C*_2_ < *C*_3_/*C*_4_, then the saturated value of PAA is controlled by the ratio of $q_{d}^{\prime }$ to *q_d_*; it is less than one if $q_{d}^{\prime }<q_{d}$ and greater than one if $q_{d}^{\prime }>q_{d}$. The saturated value is guaranteed to be less than one for $q_{d}^{\prime }<q_{d}$ if *d* is before or at the CLS since *C*_1_ is zero in this case. As shown in the Appendix, if *d* comes after the CLS, then the saturated value can still be less than one for $q_{d}^{\prime }<q_{d}$ if the output is “back-weighted” (i.e., for the steps after the CLS that go to gene product, the weighted average of the output after *d* is greater than that before *d*). Furthermore, PAA saturates to one (100%) for any value of *q_d_* and $q_{d}^{\prime }$ if *d* = 2 and *d* is before the CLS since *C*_1_ and *C*_3_ are both zero. The concentration for half maximal PAA (i.e., the EC_50_ of PAA as a function of receptor number) is given by $1/(C_{3}+C_{4}q_{d}^{\prime })$, which provides a means of assessing the relative strength of the affinity of the reactants in a downstream step, given all other things being equal.

Similarly, PP will saturate to

$$\left(\frac{C_{3}+C_{4}q_{d}}{C_{3}+C_{4}q_{d}^{\prime }})(\frac{q_{1}}{q_{1}^{\prime }}\right)$$

for large number of receptors. It will increase (decrease) to this value if $q_{d}^{\prime }<q_{d}(q_{d}^{\prime }>q_{d})$. PP is larger (smaller) if the ligand-receptor binding affinity $q_{1}^{\prime }$ is smaller (larger). If *d* = 2, the saturated value of PP is $q_{d}q_{1}/q_{d}^{\prime }q_{1}^{\prime }$. The EC_50_ of PP is the same as that for PAA. The ratio of PAA to PP is a constant for all values of receptor number. If *d* is before the CLS, then PAA/PP is $q_{d}^{\prime }q_{1}^{\prime }/q_{d}q_{1}$, which means that if the ratio of the receptor-binding affinities are known, the ratio of the downstream affinity constants can be found and *vice versa*.

More information can be obtained if we add a cofactor such as Ubc9, which has been previously shown to be an accelerator after the CLS [22]. As shown in the Appendix, the ratios can be written as

$$\begin{array}{c} \mathrm{PAA}=\left(\frac{D_{1}+\left(D_{2}+D_{3}q_{j}X_{j}^{T}\right)q_{d}^{\prime }}{D_{1}+\left(D_{2}+D_{3}q_{j}X_{j}^{T}\right)q_{d}}\right)\left(\frac{1+\left(D_{4}+\left(D_{6}+D_{8}q_{j}X_{j}^{T}\right)q_{d}\right)X_{1}^{T}}{1+\left(D_{4}+\left(D_{6}+D_{8}q_{j}X_{j}^{T}\right)q_{d}^{\prime }\right)X_{1}^{T}}\right)\\ PP=\frac{q_{1}+\left(D_{4}+\left(D_{6}+D_{8}q_{j}X_{j}^{T}\right)q_{d}\right)q_{1}X_{1}^{T}}{q_{1}^{\prime }+\left(D_{4}+\left(D_{6}+D_{8}q_{j}X_{j}^{T}\right)q_{d}^{\prime }\right)q_{1}^{\prime }X_{1}^{T}} \end{array}$$

with a different set of effective *D* parameters where *D*_1_ = 0 if *d* acts before or at the CLS and *D*_4_ = 0 if *d* = 2. Unlike the receptor, if *d* is before or at the CLS, the PAA will always saturate to 1 with added accelerator after the CLS. PAA will increase towards 1 for added accelerator for $q_{d}^{\prime }<q_{d}$ and decrease towards 1 for $q_{d}^{\prime }>q_{d}$. If PAA does not saturate to 1 with excess accelerator then *d* comes after the accelerator. PAA will also have linear fractional form if *d* acts before or at the CLS but not if *d* acts after the CLS. PP will increase (decrease) with added accelerator for $q_{d}^{\prime }<q_{d}(q_{d}^{\prime }>q_{d})$. The saturated value of PP for large amounts of accelerator is $q_{d}q_{1}/q_{d}^{\prime }q_{1}^{\prime }$. If *d* is before or at the CLS then PAA/PP is $q_{d}^{\prime }q_{1}^{\prime }/q_{d}q_{1}$ and does not depend on the accelerator but if *d* is after the CLS it will increase (decrease) with added accelerator if $q_{d}^{\prime }>q_{d}\;(q_{d}^{\prime }<q_{d})$. If PAA/PP does not change then it is the saturated value of the inverse of PP. If the accelerator is at the CLS and *d* is before the CLS then PAA and PP will not change with added accelerator. If *d* is before or at the CLS, then the EC_50_ of PAA as a function of receptor number will decrease with increasing accelerator. The amount of decrease will be larger if $q_{d}^{\prime }$ is larger. The EC_50_ of PP as a function of receptor number will have the same behavior as this independent of where *d* acts. The predictions are summarized in Tables 1 and 2.

The PAA saturates to a value of 1 with sufficient added accelerator for all antagonists that differ in affinity at a reaction before the added accelerator. If the difference is at a position after the accelerator then the saturated value of PAA will no longer be 1. Hence, the position of the difference can be probed by examining the saturated value of PAA for various added cofactors that act at different positions. Additionally, the saturated value of PAA with added accelerator provides a potential novel way to classify partial agonists. If the PAA of one partial agonist saturates to 1 while another does not, then the two partial agonists must each differ from the full agonist at a different location and can thus be considered to be in different classes.

### 3.2. Application of theory to antisteroids with changing amounts of GR and Ubc9

**Figure 1:**
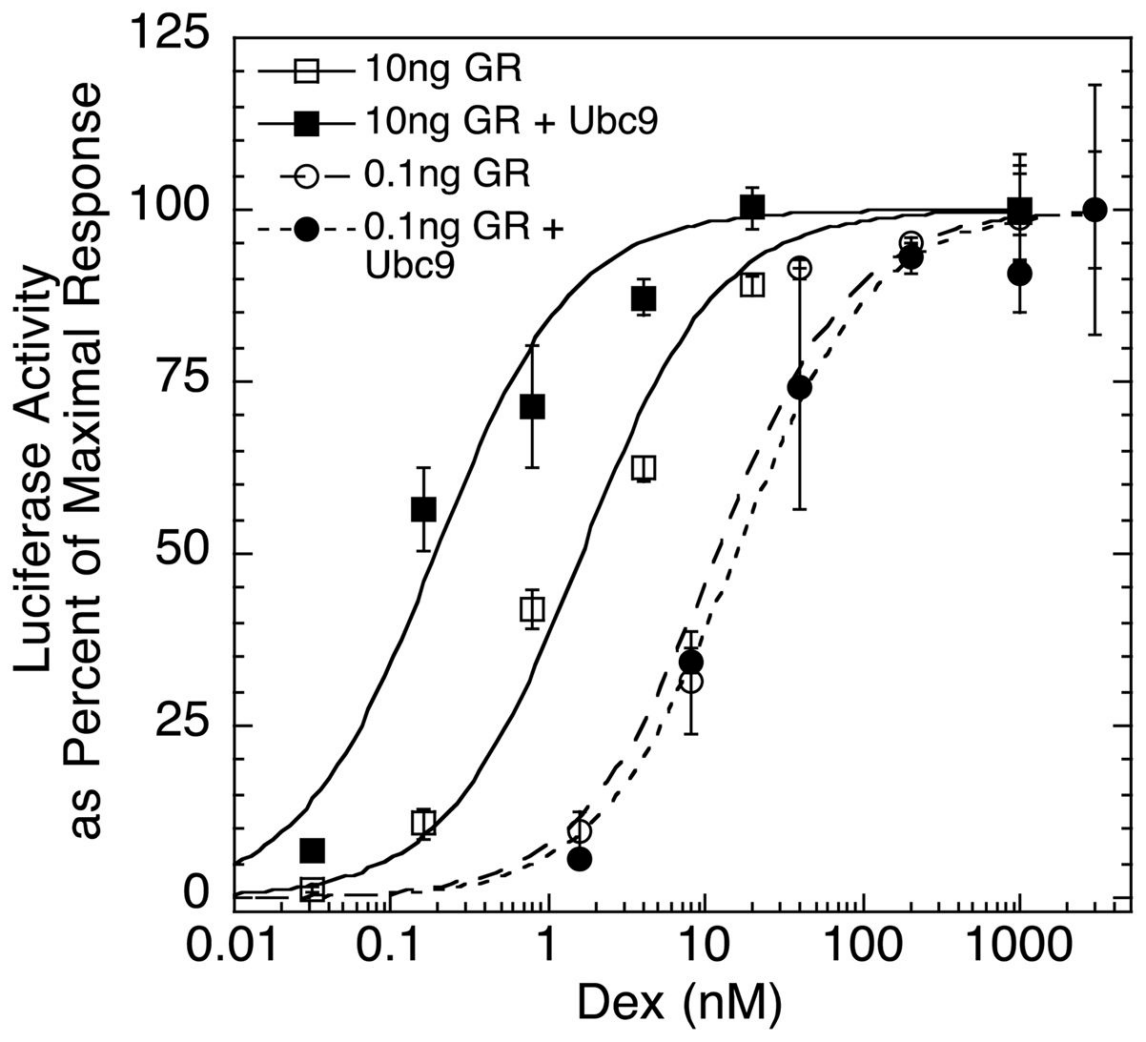
Dose-response curves of GR induction of GREtkLUC ± Ubc9 are first order Triplicate wells of CV-1 cells were transiently transfected with 100 ng of GREtkLUC reporter, 10 ng of Renilla-TS as an internal control, and the indicated amounts of pSG5/GR plasmid ± 135 ng of pSG5/Ubc9 with enough pSG5/hSA to have a constant amount of pSG5 plasmid. After 18 hr of induction by the indicated Dex concentrations, the amount of Luciferase activity in lysed cells was determined and expressed as percent of the maximal activity in each set. Error bars are S.D. of the triplicate samples. Curves are best fits for a first-order Hill plot. Similar results were obtained in three additional independent experiments.

The first requirement for the application of the theory is that the dose-response curve for GR induction of the target gene has Michaelis-Menten shape (i.e., describes a first-order Hill plot). As shown in Figure 1, Dex induction of the transfected reporter (GREtkLUC) follows a first-order Hill plot (i.e., response goes from 10% to 90% over a factor of 81 in steroid concentration) in CV-1 cells at low and high GR concentrations both with and without the added accelerator, Ubc9. Thus, we can apply the above theory to this system.

**Figure 2:**
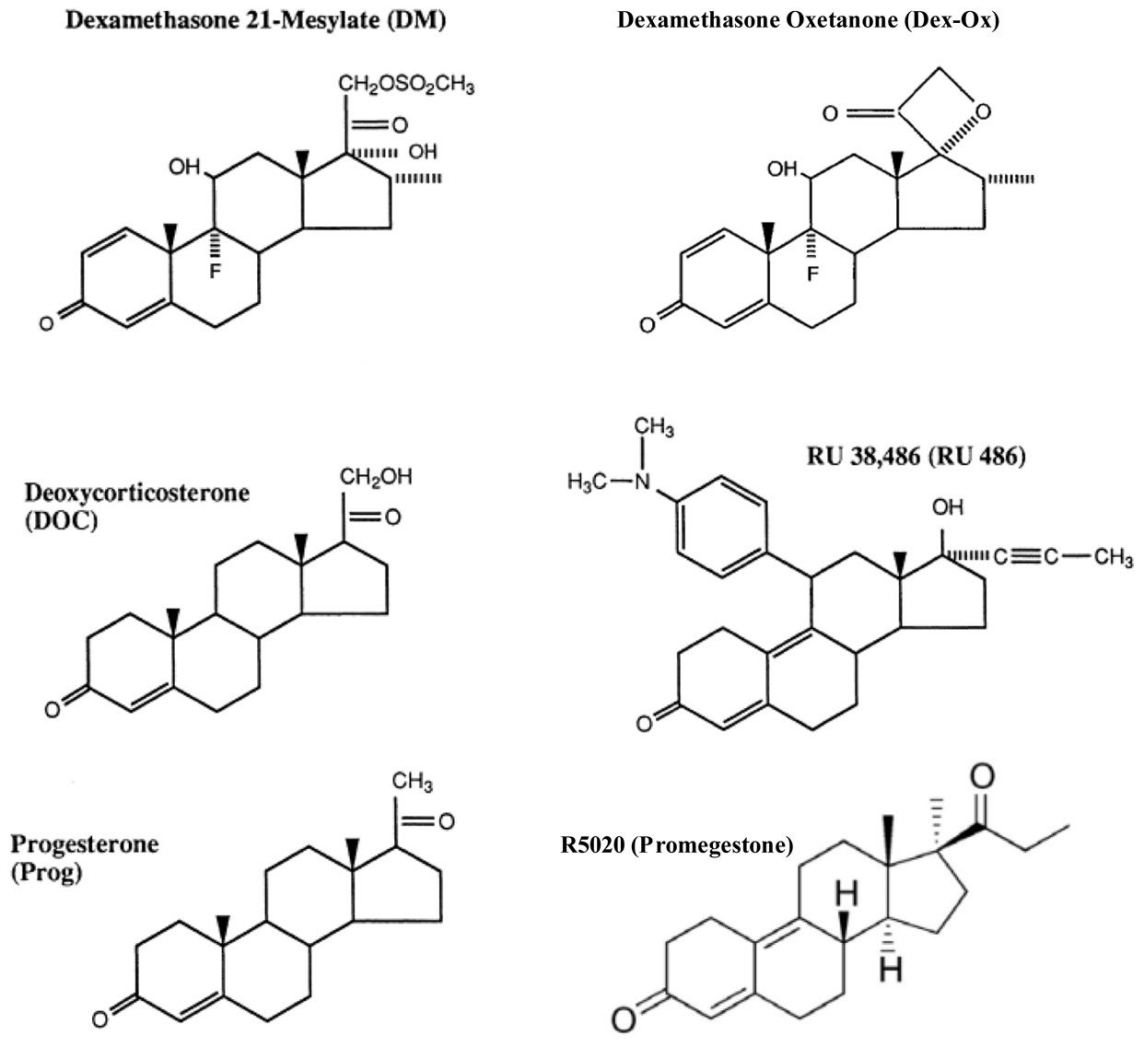
Structures of steroidal ligands used in this study.

**Figure 3:**
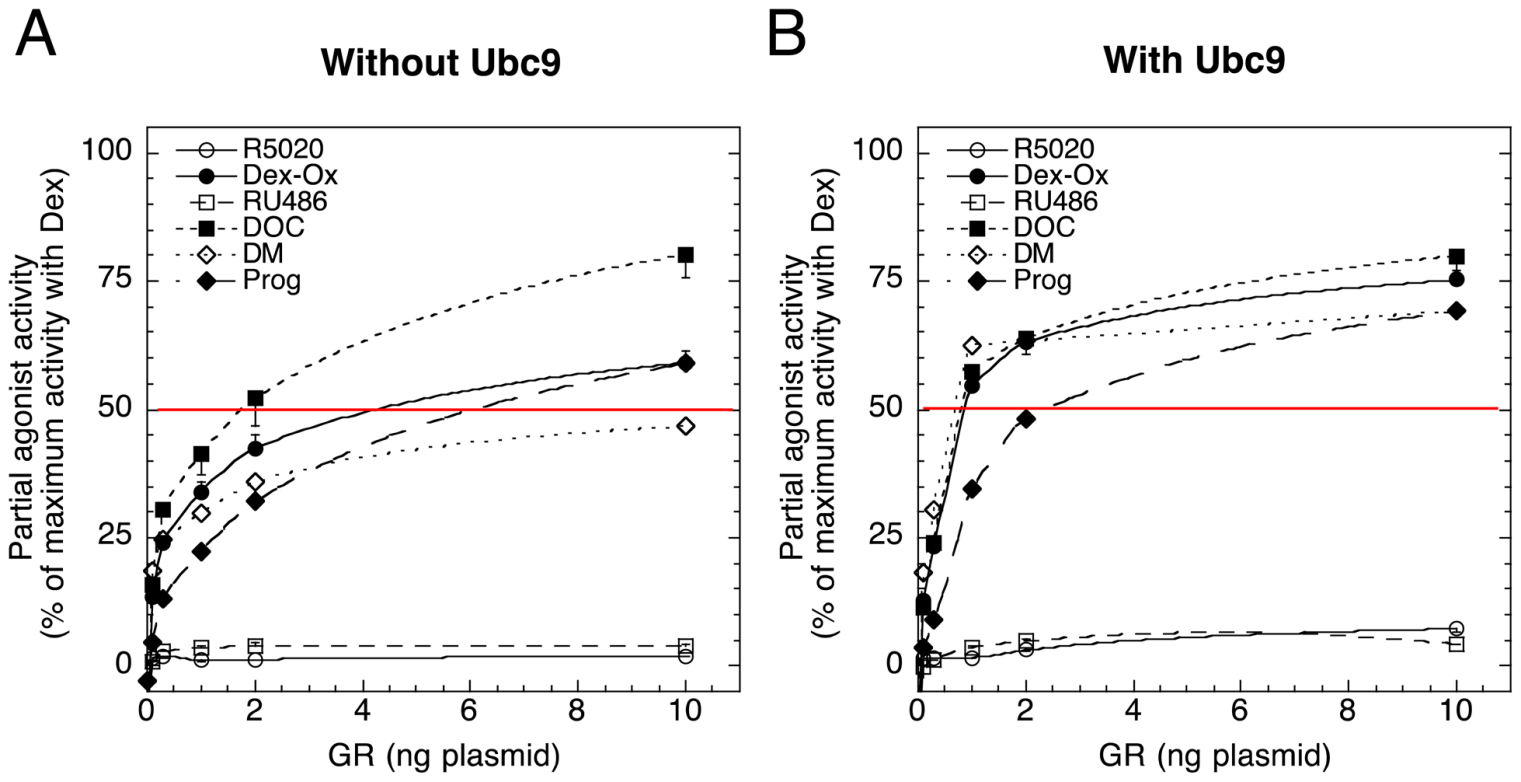
Effect of changing concentrations of GR and Ubc9 on PAA of antiglucocorticoids Experiments were conducted as in Figure 1 with the indicated amounts of pSG5/GR ± 135 ng pSG5/Ubc9 except that the steroid treatments were 1 μM antisteroid. Luciferase activities were determined and the PAA of each steroid was calculated relative to 1 μM Dex under the same conditions. The values of 4 independent experiments were averaged and plotted ± S.E.M. The thin horizontal line at 50% is only for reference.

We examined the PAA for six ligands with antiglucocorticoid activity (11-deoxycorticosterone [DOC], progesterone [Prog], R5020, RU486, dexamethasone oxetanone [Dex-Ox], and dexamethasone 21-mesylate [DM]) (Figure 2) at varying concentrations of GR and Ubc9 plasmid. As seen in Table 3, PAA as a function of receptor number was reasonably well fit by an increasing linear-fractional function (reduced *χ*^2^ was greater than but fairly close to one) both without and with added Ubc9, as predicted by the theory. At low GR (≤ 0.3 ng), added Ubc9 has little effect on the PAA, which is less than ≈ 25% (Figure 3A vs. 3B). However, added GR increases the PAA to 50–80% (Figure 3A). Importantly, Ubc9 increases the PAA even more, especially at GR concentrations of 1–2 ng (Figure 3B).

It appears that nearly full agonist activity would be achieved for DOC and Prog, (fits of Table 3 show that the 95% confidence interval for the maximum value [parameter *a*] includes 100% of full activity) with sufficient GR and Ubc9 and possibly for Dex-Ox as well, although less probable. According to the formulae as summarized in Table 1, these data predict that the difference between Dex and the antisteroids (DOC, Prog, and possibly Dex-Ox) is at a single place *d* that immediately follows steroid-receptor binding and is before or at the CLS. Despite using only one increased concentration of Ubc9, we predict that the step *d* affected by DOC, Prog, and possibly Dex-Ox must be before the position *j* of Ubc9 activity, which is known to be after the CLS [22]. Thus, according to Table 2, we confidently predict that a plot of PAA *vs*. Ubc9 would saturate to 100% for each of these antiglucocorticoids. This is consistent with the observation that added Ubc9 increases the PAA of DM in CV-1 cells to 96 ± 10% [13,33]. Table 3 shows that the EC_50_ of PAA (parameter *c*) differs for the various ligands, thus indicating that the affinity at location *d* also differs with most ligands. The EC_50_ for DOC and Prog decreased by approximately a half for increased Ubc9, as predicted by the theory, although it was not significant.

The dose-response curve for DM with increasing GR does not reach 100%. It crosses the curves for, and thus scales differently from, those for DOC, Prog, and Dex-Ox. There is also little change in the PAA of DM at GR concentrations above 2ng of plasmid. This suggests that the PAA of DM will never reach 100% with added GR. Fits to a linear-fractional function confirm that the saturated value is near 50% and significantly less than 100% (Table 3). Added Ubc9 increased the dose response so the PAA might reach 100% with sufficient Ubc9. Table 1 indicates that GR-DM complexes may differ from GR-Dex at a location after the second reaction but it is also well known that DM is an affinity label that covalently attaches to GR [34] and that the PAA of DM has been found to be inversely related to the amount of covalently bound GR formed in intact cells [16]. For example, the PAA in cells where > 90% of the GR is covalently labeled is only about 3% [15]. These data argue that DM-labeled GRs are transcriptionally inactive and that only the non-covalently labeled GRs are active. Thus, under the cellular conditions of Figure 3A where the maximum PAA of DM is about 50%, we conclude that this is because about 50% of the GR is covalently labeled and transcriptionally inactive. Conversely, with sufficient GR, the activity of the remaining 50% of GRs that are bound non-covalently by DM appear to reach a maximal activity, just as seen with DOC, Dex-Ox, and Prog, albeit at a lower value. Hence, we predict that for the 50% of non-covalently bound receptors, DM differs from Dex at the same location as DOC, Prog, and Dex-Ox. For these receptors, the difference between Dex and DM is at a single place *d*, which immediately follows the position of steroid binding to the receptor, and the affinity constant of the downstream reaction *d* with Dex is greater than that with DM. In the other 50% of the receptors, DM is forming an inactive, covalent receptor-steroid complex.

The addition of Ubc9 in Figure 3B increases the PAA with DM to 70%. This could involve Ubc9 augmenting some step *j* [22], as it does in DOC, Prog, and Dex-Ox. Alternatively, Ubc9 may rescue transactivation activity from previously inactive GR-DM complexes. This second possibility is consistent with the observations that Ubc9 was also able to dramatically increase the transcriptional activity of the otherwise almost inactive GAL/GR chimera when bound with the agonist steroid Dex [13]. It is unlikely that Ubc9 alters the labeling efficiency of GR by DM because competition assays of Ubc9 *vs.* Dex-bound GRs reveal that Ubc9 acts well downstream of GR [22], as previously proposed [33], and the same would be expected for DM-bound GRs.

RU486 is a familiar antiglucocorticoid. R5020 is less well known but does bind to GRs [35]. The PAA of both of these steroids increases to a very low value, even with high levels of GR and Ubc9. They were reasonably well fit to a linear-fractional function, although the predicted EC_50_ of the fit (parameter *c* of Table 3) was not significantly different from 0. The fact that the saturated value of PAA for both antisteroids in Fig. 3A was well below 100% is consistent with the prediction that $q_{d}^{\prime }<q_{d}$ and step *d* is either downstream of step 2 and either before or at the CLS or after the CLS and the output is back-weighted (see Table 1). PAA did increase with Ubc9 but was still significantly lower than 100%. If PAA never reaches 100% with excess Ubc9 (Figure 3B) then Table 2 indicates that the position of the affected step for each steroid is after that of Ubc9, which is after the CLS [22]. The present data do not exclude the possibility that $q_{d}^{\prime }>q_{d}$. However, it has been shown in previous experiments that there is less binding of the accelerator/coactivator GRIP1/TIF2 to DNA-bound GR-RU486 complexes than to GR-Dex complexes [12]. This lends support to the prediction that the cofactor binding affinity at step *d* may also be less with RU486-bound GRs than with Dex-bound GRs, i.e., $q_{d}^{\prime }<q_{d}$.

We predict that all of the antisteroids investigated cause the reactants of the affected step to interact with a lower affinity compared to when Dex is the ligand. A major difference, though, is that RU486 and R5020 differ in their action after the CLS (and after Ubc9) while DOC, Dex-Ox, Prog, and the active form of non-covalent GR-DM complexes all exert their actions before the CLS and at the step immediately after GR action. Either step could involve the association of corepressors/decelerators, which have not been explicitly considered in our model, but would have the effect of decreasing either the affinity or available amount of the accelerator *X^T^* at that step. However, we cannot completely exclude the possibility that with much higher (and experimentally unachievable) levels of Ubc9, the PAA of both RU486 and R5020 would approach 100%, and thus would act at the same step via the same mechanism as the other steroids in Figure 3.

## 4. Discussion

Current descriptions of antisteroid and SRM actions are unable to account for their clinically valuable properties of displaying more activity (i.e., higher PAA) with some genes than others, even in the same cell. Also unknown is why some cofactors are able to further increase or decrease the PAA of a given receptor-steroid complex with a selected gene [23,36]. We now present a general theory for SRM actions that predicts this gene-dependent modulation of PAA in ligand-regulated gene induction. Furthermore, when the PAA is modulated by a cofactor in a gene dependent manner, this theory is capable of locating the affected step relative to an invariant marker in the overall reaction sequence of GR-regulated gene transcription, i.e., the CLS [24,28]. Differential interaction of receptor-antagonist complexes with cofactors has been previously proposed [17–21] but it has not been possible to begin locating the site of modified actions before now.

The theory we have developed is predicated on the dose-response curve for gene induction being non-cooperative and described by a first-order Hill plot [22], as has been observed in the present study in CV-1 cells (Figure 1). This theory yields six major conclusions. First, the theory indicates that differences in binding affinity alone cannot account for the PAAs of antisteroids. This deduction was first unambiguously evidenced when quantitating the PAA of the affinity label, DM, which displays negligible transactivation after yielding more than 90% covalently bound receptor-steroid complex [15]. The rigorous examination of DM and other non-covalently bound antiglucocorticoids with our theory now establishes mechanistically why the PAA cannot be controlled by the affinity of any ligand for receptor.

Second, changes in PAA are controlled by differences between receptors bound by Dex and antisteroid at some other downstream step *d*. Both accelerators and decelerators (coactivators and corepressors) [24] can be accommodated by the theory, although we have specifically considered only accelerators in this study. When the PAA increases with added GR, then the affinities of the reactants at step *d* are less with the antisteroid than they are with Dex, as seen for six antagonists in Figure 3A. Furthermore, the value of the affinity of the reactants at the same step *d* that is influenced by Dex-Ox, DOC, Prog, and non-covalently bound DM is not the same but varies with steroid structure. When the PAA decreases with increasing GR, the affinity of the reactants in the presence of antagonist is greater than with Dex. We have not yet observed such a behavior but cannot eliminate the possible existence of such partial agonists.

Third, the theory requires that antisteroids directly affect a second step, downstream of steroid binding, the location of which may be probed with added cofactors. We have begun such a study here with GR and Ubc9, although it is probable that the many other known modulatory cofactors [2,8–13] will influence different steps [24,37,38] with varying efficiencies. For DOC, Dex-Ox, Prog, and non-covalently bound DM with added GR and Ubc9, we predict that the modified step is immediately after the step influenced by Dex binding to GR. It is noteworthy that Ubc9 causes a decrease, or left-shift, in the EC_50_ of the dose-response curve with both Dex [13,22,33,39] and several of the antisteroids (Table 3). The inability of elevated levels of either GR or Ubc9 to significantly alter the PAA of RU486 and R5020 is interesting and indicates action at a different step, one that is likely to be after both the CLS and site of action of Ubc9. However, we cannot completely eliminate the possibility that the tertiary structures of the receptor-steroid complexes of RU486 and R5020 may be less influenced by, or have a lower affinity for, cofactors than the other antisteroids [12]. In this case, much more GR and/or Ubc9 would be needed to reach PAA values approaching 1 or other cofactors may be more effective. It is known that the nature of the gene and the cell influence the PAA [12,36], presumably due to differences in the concentrations of assorted cofactors. Future studies with other cofactors and genes in a variety of cells should help to elucidate why some antisteroids have very low PAAs.

Fourth, it has been widely accepted that the altered structures of antisteroids perturb the topology of the resulting receptor-steroid complexes from that seen with agonists, thereby acting like a binary switch, with corepressors replacing the coactivators that associate with agonist-bound receptors [17,29]. Our present theory and data argue for replacing this switch model with a variable model. The data of Figure 3 further support our earlier postulate that cofactors, and the receptors themselves, act like a rheostat to continuously modify the PAA [9,18]. In fact, the theory not only shows how this can be accomplished but also predicts that, with enough of the appropriate factors, many if not most antisteroids will afford full agonist activity or at least attain a significant level. This prediction is supported by the data of Figure 3 for Dex-Ox, DOC, and Prog. It should be noted that the popular switch model simply represents one extreme of our continuously variable model. DM is a special case. Due to the varying amount of covalent, inactive receptor-steroid complex that is formed under different cellular conditions [16], the theoretical maximal value of PAA with DM is predicted to usually be less than 100%.

Fifth, the ability of Ubc9 to modify the PAA varies dramatically depending upon the antisteroid (Figure 3B). With other factors, the PAA of DM can be made to vary from less than 5% to over 95% [18,39]. These data suggest that very few, if any, ligands may be a full antagonist (with no PAA) under all conditions. From these data, we also predict that the ability of each antisteroid to modulate a step in the downstream interactions will not be conserved but will depend upon the steroid structure. At least part of this derives from the changes in affinity of interacting factors at the affected step *d* that can differ with steroid structure. This means that, under whole cell and whole organ conditions with limited variations in cofactor concentrations, the achievable PAA values will vary with antisteroid structure, even when the same mechanistic step is affected. Thus it should be possible to find structural modifications that will have a variety of consequences with different cells with assorted levels of numerous cofactors, not only on the PAA with a given gene but also on the PAAs with a spectrum of responsive genes. Such modularity offers tremendous possibilities for endocrine therapies in the clinical setting.

Finally, the theory predicts that an examination of the effect of cofactors on the EC_50_ of antisteroid activity will yield not only confirmation of conclusions based on PAA but also additional information about the interaction site through PP and the ratio of PAA/PP. Analyses involving antisteroid EC_50_ are technically challenging when the PAA is small, as with low GR in Figure 3, because the needed precision is more difficult to obtain with a small fold induction. However, the rewards are great with suitably responsive systems.

It is important to note that the theory we deployed here to understand partial agonist activity is identical to the theory we developed to understand steroid-regulated gene induction and repression. Here, we showed how changing receptor and cofactor concentrations could be used to make predictions for the mechanisms of antisteroids. However, the converse is also true and we could use antisteroids to probe the mechanisms of cofactors and augment our previously developed cofactor competition assay [23,27]. This new combination assay could provide information about affinity constants that would not be accessible by varying cofactor concentrations alone.

## 5. Conclusions

We have developed a theoretical model that provides new insight into the mechanism of partial agonist actions in gene induction. This theory provides a firm, theoretical framework that explains several previously observed but poorly understood phenomena, such as the role of steroid affinity for receptor, the continuously variable amounts of PAA for a given steroid/receptor/gene system, the unequal responses of antisteroids with different cofactors, and the apparent ability of many antisteroids to become full agonists under some conditions. While antisteroids, by definition, have suboptimal activity, it is now clear that this reduced activity is not fixed and can be adjusted by various cofactors in a gene- and cell-dependent manner [2,8–13]. We find that partial agonists can now be grouped on the basis of their maximal PAA with a large (albeit rarely encountered) excess of these cofactors. With all steroids examined so far, the PAA is greater with increased cofactor. In several cases, the PAA approaches 100% with added cofactor [18,21,40]. This result, predicted by the theory, indicates that the current view of antisteroids as “defective” agonists should be replaced by one where antisteroids selectively reduce the efficacy of individual downstream steps. This, in turn, provides opportunities to fine-tune gene expression in a manner that may be relatively unique for each combination of antagonist steroid, cofactor, gene, and cell type. We have used GR-regulated gene transactivation of a synthetic reporter gene in the present study. However, given the similar response of synthetic and endogenous genes in previous studies [23,36], we anticipate that the present theory will be transferable to the study of antisteroids with endogenous genes, as long as the dose-response curve is described by a first-order Hill plot. Such information with endogenous genes would be invaluable in formulating more selective endocrine therapies with antisteroids.

## Figures and Tables

**Table 1:** Predictions for changes in added receptor with downstream difference in binding affinity at location *d*.

Behavior with increasing receptor	Predictions
PAA saturates at 100%	*d* = 2 ≤ *CLS*
PAA increases to a maximal value less than 100%	$q_{d}^{\prime }<q_{d}$ and *d* ≤ *CLS* or *d* > *CLS* and output is back-weighted $q_{d}^{\prime }>q_{d}$ and *d* > *CLS* and output is front-weighted
PAA decreases to a minimal value greater than 100%	$q_{d}^{\prime }>q_{d}$ and *d* ≤ *CLS* or *d* > *CLS* and output is back-weighted $q_{d}^{\prime }<q_{d}$ and *d* > *CLS* and output is front-weighted
PP increases	$q_{d}^{\prime }<q_{d}$
PP decreases	$q_{d}^{\prime }>q_{d}$
PAA/PP does not change	Always true

**Table 2:** Predictions for changes in added accelerator at location *j* after the CLS with downstream difference in binding affinity of reaction components at location *d*.

Behavior with increasing accelerator	Predictions
PAA saturates to 100%	*d* ≤ *j*
PAA does not saturate to 100%	*d* > *j*
Saturated PAA less than 100%	$q_{d}^{\prime }<q_{d}$ and *d* > *j* and output is back-weighted $q_{d}^{\prime }>q_{d}$ and *d* > *j* and output is front-weighted
Saturated PAA greater than 100%	$q_{d}^{\prime }>q_{d}$ and *d* > *j* and output is back-weighted) $q_{d}^{\prime }<q_{d}$ and *d* > *j* and output is front-weighted
PAA increases as linear-fractional function to maximum of 100%	$q_{d}^{\prime }<q_{d}$ and *d* ≤ *CLS*
PAA decreases as linear-fractional function to minimum of 100%	$q_{d}^{\prime }>q_{d}$ and *d* ≤ *CLS*
PAA is not a linear fractional function	*d* > *CLS*
PP increases	$q_{d}^{\prime }<q_{d}$
PP decreases	$q_{d}^{\prime }>q_{d}$
PAA/PP does not change	*d* ≤ *CLS*
PAA/PP increases	*d* > *CLS* and $q_{d}^{\prime }>q_{d}$
PAA/PP decreases	*d* > *CLS* and $q_{d}^{\prime }<q_{d}$
EC_50_ of PAA as function of receptor number decreases	*d* ≤ *CLS*

**Table 3:** Parameters with 95% confidence intervals for fit to PAA = (a [ligand] + b)/(c + [ligand]), and reduced *χ*^2^ on 3 degrees of freedom For increasing PAA, parameter *a* is the saturated maximum, *b/c* is the minimum, and *c* is the EC_50_.

Without Ubc9	*a*	*b*	*c*	$\chi_{\text{red}}^{2}$
Dex Ox	56 [38, 91]	−1.8 [−10, 39]	0.39 [0.1, 3]	2.3
DOC	82 [50, 160]	1.8 [−15, 220]	0.85 [0.3, 10]	2.5
DM	40 [27, 61]	−1.0 [−5, 12]	0.11 [0, 1.5]	3.1
Prog	71 [53, 104]	1.0 [−13, 44]	2.2 [0.8, 7]	1.7
R5020	1.5 [0.65, 2.3]	0.007 [0, 0.1]	0 [−0.02, 0.02]	2.7
RU486	4.0 [2.6, 6.1]	−0.3 [−1, −0.1]	0.03 [0.006, 0.06]	2.6

With Ubc9	*a*	*b*	*c*	$\chi_{\text{red}}^{2}$
Dex Ox	79 [66, 93]	−4.0 [−7, 1]	0.41 [0.2, 0.9]	4.3
DOC	83 [70, 98]	−4.7 [−9, 0.5]	0.42 [0.2, 0.9]	2.6
DM	72 [58, 88]	−3.1 [−7, 0.4]	0.19 [0.1, 0.5]	4.0
Prog	75 [58, 97]	10.4 [−41, −.9]	0.86 [0.4, 2]	3.9
R5020	4.1 [0.6, 9]*	−0.32 [−5, 0.2]*	0.04 [−0.03, 2]*	2.4
RU486	4.3 [2.6, 6.1]	−0.5 [−1, 0.1]	0.02 [0, 0.06]	3.2

* 90% CI computed as 95% interval was not well defined.

**Table 4:** Parameter values for formulas in (A4) and (A5) where reactions 1, *i*, and, *j* obeying 1 < *i* < *j* are isolated.

	1 < *i* < *j* < cls	1 < *i* < *j* = cls	1 < *i* < cls < *j*	1 < *i* = *cls* < *j*	1 < *cls* < *i* < *j*
*B*_1_	0	0	0	0	$\Gamma_{\mathrm{cls}}^{i-1}V_{2}^{\mathrm{cls}}$
*B*_2_	0	0	$\Gamma_{\mathrm{cls}}^{j-1}V_{2}^{i-1}V_{i+1}^{\mathrm{cls}}$	$\Gamma_{\mathrm{cls}}^{j-1}V_{2}^{\mathrm{cls}-1}$	$\Gamma_{i}^{j-1}V_{2}^{i-1}$
*B*_3_	$\Gamma_{\mathrm{cls}}^{n}V_{2}^{i-1}V_{i+1}^{j-1}V_{j+1}^{\mathrm{cls}}$	$\Gamma_{\mathrm{cls}}^{n}V_{2}^{i-1}V_{i+1}^{\mathrm{cls}-1}$	$\Gamma_{j}^{n}V_{2}^{i-1}V_{i+1}^{j-1}$	$\Gamma_{j}^{n}V_{2}^{\mathrm{cls}-1}V_{\mathrm{cls}+1}^{j-1}$	$\Gamma_{j}^{n}V_{2}^{i-1}V_{i+1}^{j-1}$
*B*_4_	$W_{2}^{i-1}$	$W_{2}^{i-1}$	$W_{2}^{i-1}$	$W_{2}^{\mathrm{cls}-1}$	$W_{2}^{\mathrm{cls}-1}+q_{\mathrm{cls}}V_{2}^{\mathrm{cls}-1}\sum_{k=\mathrm{cls}}^{i-1}V_{\mathrm{cls}+1}^{k}$
*B*_5_	$V_{2}^{i-1}$	$V_{2}^{i-1}$	$V_{2}^{i-1}$	0	0
*B*_6_	$V_{2}^{i-1}W_{i+1}^{j-1}$	$V_{2}^{i-1}W_{i+1}^{\mathrm{cls}-1}$	$V_{2}^{i-1}W_{i+1}^{\mathrm{cls}-1}+q_{\mathrm{cls}}V_{2}^{i-1}V_{i+1}^{\mathrm{cls}-1}\sum_{k=\mathrm{cls}}^{j-1}V_{\mathrm{cls}+1}^{k}$	$V_{2}^{\mathrm{cls}-1}\sum_{k=\mathrm{cls}}^{j-1}V_{\mathrm{cls}+1}^{k}$	$q_{\mathrm{cls}}V_{2}^{\mathrm{cls}-1}V_{\mathrm{cls}+1}^{i-1}\sum_{k=i}^{j-1}V_{i+1}^{k}$
*B*_7_	$V_{2}^{i-1}V_{i+1}^{j-1}$	$V_{2}^{i-1}V_{i+1}^{\mathrm{cls}-1}\sum_{k=\mathrm{cls}}^{n}V_{\mathrm{cls}+1}^{k}$	0	0	0
*B*_8_	$V_{2}^{i-1}V_{i+1}^{j-1}W_{j+1}^{\mathrm{cls}-1}+q_{\mathrm{cls}}V_{2}^{i-1}V_{i+1}^{j-1}V_{j+1}^{\mathrm{cls}-1}\sum_{k=\mathrm{cls}}^{n}V_{\mathrm{cls}+}^{k}$	0	$q_{\mathrm{cls}}V_{2}^{i-1}V_{i+1}^{\mathrm{cls}-1}V_{\mathrm{cls}+1}^{j-1}\sum_{k=j}^{n}V_{j+}^{k}$	$V_{2}^{\mathrm{cls}-1}V_{\mathrm{cls}+1}^{j-1}\sum_{k=j}^{n}V_{j+1}^{k}$	$q_{\mathrm{cls}}V_{2}^{\mathrm{cls}-1}V_{\mathrm{cls}+1}^{i-1}V_{i+1}^{j-1}\sum_{k=j}^{n}V_{j+1}^{k}$
*λ*	1	1	1	0	1

## Appendix 1. Derivation of formulae

Consider the biochemical reaction sequence $Y_{i-1}+X_{i}\overset{q_{i}}{↔}Y_{i}$, where *i* = 1, 2, 3,…, *n*, *X*_i_ are accelerators, *Y*_i_ are products, and *q*_i_ are equilibrium or affinity constants (i.e., ratio of forward to backward rate constants). Each step can also be inhibited by decelerators [22,23,28]. Although, we do not consider them here, they can be easily incorporated into the formulae. Each reaction is reversible and thus obeys detailed balance in equilibrium. In the mass action, mean field limit, the concentrations obey equilibrium conditions [*Y_i_*] = *q_i_*[*X_i_*][*Y_i_*_−1_] and mass conservation conditions $[X_{i}]+[Y_{i}]+\cdots +[Y_{n}]=X_{i}^{T}$, where the square bracket denotes a concentration (i.e., molecules per unit volume) and $X_{i}^{T}$ is the total concentration of *X_i_* . For a single reaction, the two conditions are [*Y*_1_] = *q*_1_[*X*_1_][*Y*_0_] and $[X_{1}]+[Y_{1}]=X_{1}^{T}$. We can solve these two equations in three unknowns for [*Y*_1_] as a function of [*Y*_0_] to obtain the non-cooperative Michaelis-Menten dose-response curve

(A1) $$[Y_{1}]=\frac{q_{1}X_{1}^{T}\;[Y_{0}]}{1+q_{1}\;[Y_{0}]}$$

Note that the A_max_ of (A1) is $X_{1}^{T}$ and does not depend on the binding affinity. This gives a simple explanation for why receptor-ligand binding affinity cannot change the dose-response. For two reactions, the equilibrium and mass conservation equations are

$$\begin{array}{l} \left[Y_{1}]=q_{1}\;[X_{1}]\;[Y_{0}\right]\\ \left[Y_{2}]=q_{2}\;[X_{2}]\;[Y_{1}\right] \end{array}$$

$$\begin{array}{r} {}[X_{1}]+[Y_{1}]+[Y_{2}]=X_{1}^{T}\\ {}[X_{2}]+[Y_{2}]=X_{2}^{T} \end{array}$$

However, as shown previously [22,28], these equations do not generally lead to a non-cooperative dose-response for [*Y*_2_] as a function of [*Y*_0_]. If however, [*Y*_2_] has a small concentration compared to [*X*_1_] and [*Y*_1_] then it can be eliminated in the first mass conservation equation and the reactions effectively decouple. We then obtain (A1) and

(A2) $$[Y_{2}]=\frac{q_{2}X_{2}^{T}\;[Y_{1}]}{1+q_{2}\;[Y_{1}]}$$

Substituting [*Y*_1_] from (A1) into (A2) yields the dose-response

(A3) $$[Y_{2}]=\frac{q_{1}X_{1}^{T}q_{2}X_{2}^{T}\;[Y_{0}]}{1+\left(q_{1}+q_{1}X_{1}^{T}q_{2}\right)[Y_{0}]}$$

which preserves the Michaelis-Menten shape. This property occurs because Michaelis-Menten functions are in the family of linear-fractional functions, which form a group under function composition. If concentrations of downstream products are low (or lifetimes are short) compared to upstream products then all reactions effectively decouple [22,28] and each product is a Michaelis-Menten function of the previous product. The reactions can also decouple if accelerators are superabundant compared to products. The group property then ensures that the dose-response is also Michaelis-Menten.

Given the group property, the function between two adjacent products

$$[Y_{i+1}]=\frac{q_{i}X_{i}^{T}\;[Y_{i}]}{1+q_{i}\;[Y_{i}]}$$

can be represented by the matrix

$$M_{i}=\left(\begin{array}{cc} v_{i} & 0\\ w_{i} & 1 \end{array}\right)$$

where $v_{i}=q_{i}X_{i}^{T}$ is the A_max_ / EC_50_ and *w_i_* = *q_i_* is the 1/EC_50_ of [*Y*_i_]. All matrices before the CLS have this form but at the CLS, *w_i_* is replaced by $q_{\mathrm{cls}}\left(1+\sum_{k=\mathrm{cls}+1}^{n}\prod_{i=\mathrm{cls}+1}^{k}v_{i}\right)$, and after the CLS, *w_i_* = 0. Both *v_i_* and *w_i_* are modified in the presence of decelerators [22,23,28].

Substituting one dose-response formula into another is represented by matrix multiplication. For example, A_max_/EC_50_ and 1/EC_50_ for (A3) are obtained directly from the upper left and lower left elements of the matrix *M*_2_*M*_1_ . The group property also implies that any product of matrices telescopes down into a lower triangular matrix of the same form. The product of matrices from step *i* to *j* can be written as

$$P_{i}^{j}=\left(\begin{array}{cc} V_{i}^{j} & 0\\ W_{i}^{j} & 1 \end{array}\right)$$

where $V_{i}^{j}=\prod_{k=i}^{j}v_{k}$ and $W_{i}^{j}=\sum_{k=i}^{j}w_{k}V_{i}^{k-1}$, and we use the convention that $V_{j+1}^{j}=1,\;V_{i}^{j}=0$, *j*<*i*−1, and $W_{i}^{j}=0$, *j*<*i*. For *n* reactions, the output of the last reaction as a function of the agonist is given by $P_{1}^{n}$. Suppose we are interested in understanding how the parameters in three of the reactions (1, *i*, and *j*) affect this output. The product can be decomposed into $P_{1}^{n}=P_{j+1}^{n}M_{j}P_{i+1}^{j-1}M_{i}P_{2}^{i-1}M_{1}$, where the elements of the *P* matrices are made of the parameters from all the intervening reactions but can be treated as effective nonnegative parameters. However, this is not the most general form for the dose-response. Since all matrices *M*_i_ after the CLS are diagonal with 1 in the lower right corner, multiplication by a post-CLS matrix only modifies the upper left entry. This means that the dose-response function for every post-CLS product has the same denominator. Hence, the concentrations of all these products can be directly summed and still preserve Michaelis-Menten shape. Biochemically, this means that each step at and after the CLS could go directly to the gene product and the dose response would have the same shape. Thus the total product can be written as a weighted sum over the outputs of each of these steps. Mathematically, the Michaelis-Menten shape is preserved by two independent algebraic structures. One is the group of lower triangular matrices, which represents function composition of Michaelis-Menten functions, and the other is a vector space where fractions with the same denominator can be summed to preserve the denominator. Therefore, the general form of the dose-response can be written as

(A4) $$[Y_{n}]=\frac{\Gamma_{\mathrm{cls}}^{n}V_{1}^{\mathrm{cls}}[Y_{0}]}{1+W_{l}^{\mathrm{cls}}[Y_{0}]}$$

where $\Gamma_{l}^{m}=\sum_{k=l}^{m}a_{k-\mathrm{cls}}V_{l+1}^{k},\;A_{\max}/{EC}_{50}=\Gamma_{\mathrm{cls}}^{n}V_{1}^{\mathrm{cls}}$, and $1/{EC}_{50}=W_{1}^{\mathrm{cls}}$.

We construct formulae for PAA and PP from the dose response in (A4) by decomposing A_max_/EC_50_ and 1/EC_50_ to isolate the influence of 3 reactions: 1) The first reaction where the ligand binds to the receptor, 2) a reaction downstream of reaction 1 where the affinity constant differs between agonists, and 3) a third reaction where an added cofactor acts. The most general form for A_max_/EC_50_ in terms of *q_1_*, *q_i_*, *q_j_*, $X_{1}^{T}$, and $X_{j}^{T}$ for 1<*i*<*j* can be expressed as [22,23,28]

(A5) $$\frac{A_{\max}}{{EC}_{50}}=\left(B_{1}+\left(B_{2}+B_{3}q_{j}X_{j}^{T}\right)q_{i}X_{i}^{T}\right)q_{1}X_{1}^{T}$$

where the nonnegative *B* parameters take on different forms depending on the relative positions of *i* and *j* with respect to the CLS and are listed in Table 4 for all the relevant cases. For example, parameter *B*_1_ = 0 if step *i* is before or at the CLS and *B*_1_ = *B*_2_ = 0 if step *j* is before or at the CLS. Also, *B*_1_ and *B*_2_ get larger as *j* moves downstream with respect to the CLS. The most general form for 1/EC_50_ is [22,23,28]

(A6) $$\frac{1}{{EC}_{50}}=q_{1}+\left(B_{4}+B_{5}q_{i}+\left(B_{6}+B_{7}q_{j}+B_{8}q_{j}X_{j}^{T}\right)q_{i}\left[1-\lambda +\lambda X_{i}^{T}\right]\right)q_{i}X_{1}^{T}$$

where the parameters are listed in Table 4. Noteworthy from the table is that the total accelerator concentrations $X_{i}^{T}$ or $X_{j}^{T}$ do not appear in the formula if *i* or *j* is at the CLS. In other words, EC_50_ is unaffected by an accelerator at the CLS, as described in [24]. Also, *B*_4_ = 0 if *i* = 2 and increases monotonically for *i*≤*CLS*.

The PAA and PP follow from (A5) and (A6) and have the forms:

$$\begin{array}{c} \mathrm{PAA}=\left(\frac{B_{1}+\left(B_{2}+B_{3}q_{j}^{\prime }X_{j}^{T}\right)q_{i}^{\prime }X_{i}^{T}}{B_{1}+\left(B_{2}+B_{3}q_{j}X_{j}^{T}\right)q_{i}X_{i}^{T}}\right)\left(\frac{1+\left(B_{4}+B_{5}q_{i}+\left(B_{6}+B_{7}q_{j}+B_{8}q_{j}X_{j}^{T}\right)q_{i}\left[1-\lambda +\lambda X_{i}^{T}\right]\right)X_{1}^{T}}{1+\left(B_{4}+B_{5}q_{i}^{\prime }+\left(B_{6}+B_{7}q_{j}+B_{8}q_{j}^{\prime }X_{j}^{T}\right)q_{i}^{\prime }\left[1-\lambda +\lambda X_{i}^{T}\right]\right)X_{1}^{T}}\right)\\ PP=\frac{q_{1}+\left(B_{4}+B_{5}q_{i}+\left(B_{6}+B_{7}q_{j}+B_{8}q_{j}X_{j}^{T}\right)q_{i}\left[1-\lambda +\lambda X_{i}^{T}\right]\right)q_{1}X_{1}^{T}}{q_{1}^{\prime }+\left(B_{4}+B_{5}q_{i}^{\prime }+\left(B_{6}+B_{7}q_{j}^{\prime }+B_{8}q_{j}^{\prime }X_{j}^{T}\right)q_{i}^{\prime }\left[1-\lambda +\lambda X_{i}^{T}\right]\right)q_{1}^{\prime }X_{1}^{T}} \end{array}$$

and the ratio of the two is given by

$$\frac{\mathrm{PAA}}{PP}=\left(\frac{B_{1}+\left(B_{2}+B_{3}q_{j}^{\prime }X_{j}^{T}\right)q_{i}^{\prime }X_{i}^{T}}{B_{1}+\left(B_{2}+B_{3}q_{j}X_{j}^{T}\right)q_{i}X_{i}^{T}}\right)\frac{q_{1}^{\prime }}{q_{1}}$$

If we are only interested in how these ratios change with respect to the added receptor $X_{1}^{T}$ and the action of the partial and full agonist only differs in affinity at site *d*, then we can simplify the formulae in terms of a new set of effective parameters. Setting *i* = *d*, *C*_1_ = *B*_1_, $C_{2}=\left(B_{2}+B_{3}q_{j}X_{j}^{T}\right)X_{d}^{T}$, *C*_3_ = *B*_4_, and $C_{4}=B_{5}+\left(B_{6}+B_{7}q_{j}+B_{8}q_{j}X_{j}^{T}\right)\left[1-\lambda +\lambda X_{i}^{T}\right]$, gives the equations in the main text.

Predictions on the position of *d* and the relative strengths of the affinities at *d* depend on the form of these *C* parameters. From Table 4 we see that if *d* is before or at the CLS then *C*_1_ = 0 and $C_{3}=\sum_{k=2}^{d-1}q_{k}V_{2}^{k-1}$. Hence *C* = 0 if *d* = 2. If *d* is after the CLS then $C_{1}=\Gamma_{\mathrm{cls}}^{d-1}V_{2}^{\mathrm{cls}},\;C_{2}=\Gamma_{\mathrm{cls}}^{n}V_{2}^{\mathrm{cls}}V_{\mathrm{cls}+1}^{d-1}X_{d}^{T},\;C_{3}=\sum_{k=2}^{\mathrm{cls}-1}q_{k}V_{2}^{k-1}+q_{\mathrm{cls}}V_{2}^{\mathrm{cls}-1}\sum_{k=\mathrm{cls}}^{d-1}V_{\mathrm{cls}+1}^{k}$, and $C_{4}=\left(q_{\mathrm{cls}}V_{2}^{\mathrm{cls}-1}V_{\mathrm{cls}+1}^{d-1}\sum_{k=i}^{n}V_{d+1}^{k}\right)X_{d}^{T}$, where we have set $q_{j}^{\prime }=q_{j}$. Hence,

$$\begin{array}{c} C_{1}/C_{2}=\left(\sum_{k=\mathrm{cls}}^{d-1}a_{k-\mathrm{cls}}V_{\mathrm{cls}+1}^{k}\right)/\left(V_{\mathrm{cls}+1}^{d-1}X_{d}^{T}\sum_{k=d}^{n}a_{k-\mathrm{cls}}V_{d+1}^{k}\right)\\ C_{3}/C_{4}=\left(\sum_{k=2}^{\mathrm{cls}-1}q_{k}V_{2}^{k}/q_{\mathrm{cls}}V_{2}^{\mathrm{cls}-1}+\sum_{k=\mathrm{cls}}^{d-1}V_{\mathrm{cls}+1}^{k}\right)/\left(V_{\mathrm{cls}+1}^{d-1}X_{d}^{T}\sum_{k=d}^{n}V_{d+1}^{k}\right) \end{array}$$

*C*_1_/*C*_2_ and *C*_3_/*C*_4_ have similar forms except that the sums in *C*_1_/*C*_2_ are weighted by the nonnegative *a* parameters. Since we can divide the numerator and denominator of *C*_1_/*C*_2_ by the maximum *a* parameter, only the relative magnitudes determine if *C*_1_/*C*_2_ or *C*_3_/*C*_4_ is larger. *C*_1_/*C*_2_ is less than *C*_3_/*C*_4_ if the *a* parameters are uniform or “back-weighted” (i.e. if the average of the *a* parameters after *d* is greater than or equal to the average before *d*). Analogously, if we are interested in the action of an accelerator, $X_{j}^{T}$ after the CLS then we can write PAA and PP in terms of a new set of effective *D* parameters as seen in the main text. Similar analyses of the properties of the ratios can also be performed.

## References

[1] Zajchowski DA, Kauser K, Zhu D (2000). Identification of selective estrogen receptor modulators by their gene expression fingerprints. J Biol Chem.

[2] Simons SS (2003). The importance of being varied in steroid receptor transactivation. TIPS.

[3] Johnson AB, O’Malley BW (2012). Steroid receptor coactivators 1, 2, and 3: Critical regulators of nuclear receptor activity and steroid receptor modulator (SRM)-based cancer therapy. Mol Cell Endocrinol.

[4] Shang Y, Brown M (2002). Molecular determinants for the tissue specificity of SERMs. Science.

[5] Zalachoras I, Houtman R, Atucha E (2013). Differential targeting of brain stress circuits with a selective glucocorticoid receptor modulator. Proc Natl Acad Sci U S A.

[6] MacGregor JI, Jordan VC (1998). Basic guide to the mechanisms of antiestrogen action. Pharmacol Rev.

[7] Ojasoo T, Dore J-C, Gilbert J, Raynaud J-P (1988). Binding of steroids to the progestin and glucocorticoid receptors analyzed by correspondence analysis. J Med Chem.

[8] Szapary D, Xu M, Simons SS (1996). Induction properties of a transiently transfected glucocorticoid-responsive gene vary with glucocorticoid receptor concentration. J Biol Chem.

[9] Wang Q, Blackford JA, Song L-N (2004). Equilibrium interactions of corepressors and coactivators modulate the properties of agonist and antagonist complexes of glucocorticoid receptors. Mol Endocrinol.

[10] Simons SS, Edwards DP, Kumar R (2014). Minireview: dynamic structures of nuclear hormone receptors: new promises and challenges. Mol Endocrinol.

[11] Simons SS (2008). What goes on behind closed doors: physiological versus pharmacological steroid hormone actions. Bioessays.

[12] Cho S, Blackford JA, Simons SS (2005). Role of activation function domain 1, DNA binding, and coactivator in the expression of partial agonist activity of glucocorticoid receptor complexes. Biochemistry.

[13] Cho S, Kagan BL, Blackford JA (2005). Glucocorticoid receptor ligand binding domain is sufficient for the modulation of glucocorticoid induction properties by homologous receptors, coactivator transcription intermediary factor 2, and Ubc9. Mol Endo.

[14] Raynaud JP, Bouton MM, Ojasoo T (1980). The use of interaction kinetics to distinguish potential antagonists from agonists. TIPS.

[15] Sistare FD, Hager GL, Simons SS (1987). Mechanism of dexamethasone 21-mesylate antiglucocorticoid action: I. Receptor-antiglucocorticoid complexes do not competitively inhibit receptor-glucocorticoid complex activation of gene transcription in vivo. Mol Endocrinol.

[16] Miller PA, Simons SS (1988). Comparison of glucocorticoid receptors in two rat hepatoma cell lines with different sensitivities to glucocorticoids and antiglucocorticoids. Endocrinology.

[17] Nagy L, Schwabe JW (2004). Mechanism of the nuclear receptor molecular switch. Trends Biochem Sci.

[18] Szapary D, Huang Y, Simons SS (1999). Opposing effects of corepressor and coactivators in determining the dose-response curve of agonists, and residual agonist activity of antagonists, for glucocorticoid receptor regulated gene expression. Mol Endocrinol.

[19] Shiau AK, Barstad D, Radek JT (2002). Structural characterization of a subtype-selective ligand reveals a novel mode of estrogen receptor antagonism. Nat Struct Biol.

[20] Tao Y-G, Xu Y, Xu HE (2008). Mutations of glucocorticoid receptor differentially affect AF2 domain activity in a steroid-selective manner to alter the potency and efficacy of gene induction and repression. Biochemistry.

[21] Lee G-S, Simons SS (2011). Ligand binding domain mutations of glucocorticoid receptor selectively modify effects with, but not binding of, cofactors. Biochemistry.

[22] Ong KM, Blackford JA, Kagan BL (2010). A theoretical framework for gene induction and experimental comparisons. Proc Natl Acad Sci U S A.

[23] Dougherty EJ, Guo C, Simons SS (2012). Deducing the temporal order of cofactor function in ligand-regulated gene transcription: theory and experimental verification. PLoS ONE.

[24] Blackford JA, Guo C, Zhu R (2012). Identification of Location and Kinetically Defined Mechanism of Cofactors and Reporter Genes in the Cascade of Steroid-regulated Transactivation. J Biol Chem.

[25] Luo M, Lu X, Zhu R (2013). A Conserved Protein Motif Is Required for Full Modulatory Activity of Negative Elongation Factor Subunits NELF-A and NELF-B in Modifying Glucocorticoid Receptor-regulated Gene Induction Properties. J Biol Chem.

[26] Zhang Z, Sun Y, Cho Y-W (2013). PA1: a new competitive decelerator acting at more than one step to impede glucocorticoid receptor-mediated transactivation. J Biol Chem.

[27] Zhu R, Lu X, Pradhan M (2014). A kinase-independent activity of Cdk9 modulates glucocorticoid receptor-mediated gene induction. Biochemistry.

[28] Chow CC, Finn KK, Storchan GBL (2015). Kinetically-defined component actions in gene repression. PLoS Comput Biol.

[29] Perissi V, Rosenfeld MG (2005). Controlling nuclear receptors: the circular logic of cofactor cycles. Nat Rev Mol Cell Biol.

[30] Pons M, Simons SS (1981). Facile, high yield synthesis of spiro C-17-steroidal oxetan-3′-ones. J Org Chem.

[31] Kaul S, Blackford JA, Chen J (2000). Properties of the glucocorticoid modulatory element binding proteins GMEB-1 and -2: potential new modifiers of glucocorticoid receptor transactivation and members of the family of KDWK proteins. Mol Endocrinol.

[32] He Y, Simons SS (2007). STAMP: a novel predicted factor assisting TIF2 actions in glucocorticoid receptor-mediated induction and repression. Mol Cell Biol.

[33] Kaul S, Blackford JA, Cho S (2002). Ubc9 is a novel modulator of the induction properties of glucocorticoid receptors. J Biol Chem.

[34] Simons SS, Thompson EB (1981). Dexamethasone 21-mesylate: an affinity label of glucocorticoid receptors from rat hepatoma tissue culture cells. Proc Natl Acad Sci USA.

[35] Stromstedt P-E, Berkenstam A, Jornvall H (1990). Radiosequence analysis of the human progestin receptor charged with [3H]promegestone. A comparison with the glucocorticoid receptor. J Biol Chem.

[36] Luo M, Simons SS (2009). Modulation of glucocorticoid receptor induction properties by cofactors in peripheral blood mononuclear cells. Hum Immunol.

[37] Blackford JA, Brimacombe KR, Dougherty EJ (2014). Modulators of glucocorticoid receptor activity identified by a new high-throughput screening assay. Mol Endocrinol.

[38] Simons SS, Kumar R (2013). Variable steroid receptor responses: Intrinsically disordered AF1 is the key. Mol Cell Endocrinol.

[39] Kim Y, Sun Y, Chow C (2006). Effects of acetylation, polymerase phosphorylation, and DNA unwinding in glucocorticoid receptor transactivation. J Steroid Biochem Molec Biol.

[40] Giannoukos G, Szapary D, Smith CL (2001). New antiprogestins with partial agonist activity: potential selective progesterone receptor modulators (SPRMs) and probes for receptor- and coregulator-induced changes in progesterone receptor induction properties. Mol Endocrinol.

